# Self-Reported Fatigue After Mild Traumatic Brain Injury Is Not Associated With Performance Fatigability During a Sustained Maximal Contraction

**DOI:** 10.3389/fphys.2018.01919

**Published:** 2019-01-10

**Authors:** Roeland F. Prak, Joukje van der Naalt, Inge Zijdewind

**Affiliations:** ^1^Department of Biomedical Sciences of Cells and Systems, University Medical Center Groningen, University of Groningen, Groningen, Netherlands; ^2^Department of Neurology, University Medical Center Groningen, University of Groningen, Groningen, Netherlands

**Keywords:** mTBI, motor task, force decline, voluntary muscle activation, interpolated twitch technique, first dorsal interosseous

## Abstract

Patients with mild traumatic brain injury (mTBI) are frequently affected by fatigue. However, hardly any data is available on the fatigability of the motor system. We evaluated fatigue using the Fatigue Severity Scale (FSS) and Modified Fatigue Impact Scale (MFIS) questionnaires in 20 participants with mTBI (>3 months post injury; 8 females) and 20 age- and sex matched controls. Furthermore, index finger abduction force and electromyography of the first dorsal interosseous muscle of the right hand were measured during brief and sustained maximal voluntary contractions (MVC). Double pulse stimulation (100 Hz) was applied to the ulnar nerve to evoke doublet-forces before and after the sustained contraction. Seven superimposed twitches were evoked during the sustained MVC to quantify voluntary muscle activation. mTBI participants reported higher FSS scores (mTBI: 5.2 ± 0.8 *SD* vs. control: 2.8 ± 0.8 *SD*; *P* < 0.01). During the sustained MVC, force declined to similar levels in mTBI (30.0 ± 9.9% MVC) and control participants (32.7 ± 9.8% MVC, *P* = 0.37). The decline in doublet-forces after the sustained MVC (mTBI: to 37.2 ± 12.1 vs. control: to 41.4 ± 14.0% reference doublet, *P* = 0.32) and the superimposed twitches evoked during the sustained MVC (mTBI: median 9.3, range: 2.2–32.9 vs. control: median 10.3, range: 1.9–31.0% doublet_pre_, *P* = 0.34) also did not differ between groups. Force decline was associated with decline in doublet-force (*R*^2^ = 0.50, *P* < 0.01) for both groups. Including a measure of voluntary muscle activation resulted in more explained variance for mTBI participants only. No associations between self-reported fatigue and force decline or voluntary muscle activation were found in mTBI participants. However, the physical subdomain of the MFIS was associated with the decline in doublet-force after the sustained MVC (*R*^2^ = 0.23, *P* = 0.04). These results indicate that after mTBI, increased levels of self-reported physical fatigue reflected increased fatigability due to changes in peripheral muscle properties, but not force decline or muscle activation. Additionally, muscle activation was more important to explain the decline in voluntary force (performance fatigability) after mTBI than in control participants.

## Introduction

Traumatic brain injury is a serious health problem and one of the most important causes of impairment in adults; most cases sustain a mild traumatic brain injury (mTBI; Cassidy et al., 2014; Levin and Diaz-Arrastia, 2015). Despite the fact that most patients recover from mTBI, 15–20% of patients have persistent symptoms (Cassidy et al., 2014; Levin and Diaz-Arrastia, 2015) and fatigue is among the most frequently occurring complaints (van der Naalt et al., 1999; Lundin et al., 2006; Lannsjö et al., 2009; Mollayeva et al., 2014). Fatigue is a disabling symptom because it impairs physical and social functioning, and it impedes return to work (van der Naalt et al., 1999, 2017; Stulemeijer et al., 2006; de Koning et al., 2017).

Nowadays, the term fatigue is most often reserved for the self-reported symptom which can be evaluated using questionnaires. According to the taxonomy proposed by Kluger and colleagues, fatigue is mediated by two attributes (Kluger et al., 2013). The first attribute, perceived fatigability, corresponds to changes in subjective sensations occurring at rest or in the context of task performance and derives from psychological and homeostatic factors (Enoka and Duchateau, 2016). The second attribute, performance fatigability, relates to impaired task performance and is defined as the decline in an objective measure of performance over a discrete period of time (e.g., a decline in voluntary force or power during sustained contractions; Enoka and Duchateau, 2016). Although perceived and performance fatigability are distinct qualities they can interact.

So far, most studies investigating fatigue following mTBI have focussed on the relationship between self-reported fatigue and cognitive task performance (Möller et al., 2014, 2017; Nordin et al., 2016; Wylie and Flashman, 2017). Although the impact of mTBI on cognitive parameters is prominent, few groups also report (long-lasting) impairments in motor parameters including: a small reduction in voluntary drive (Powers et al., 2014), inhibitory changes in the primary motor cortex (De Beaumont et al., 2009), and reduced walking speed in a dual-task condition (Yasen et al., 2017). However, no data is available on the fatigability of the motor system in mTBI.

Another neurological patient population suffering from fatigue is multiple sclerosis (MS). Previous studies by our lab showed that self-reported fatigue in persons with MS was associated with measures of performance fatigability during a sustained maximal contraction These studies provided the rationale and experimental framework to investigate fatigue following mTBI (Steens et al., 2012; Wolkorte et al., 2015a). Although differences in the pathophysiology of MS and mTBI are evident, the conditions have aspects in common including neuroinflammation and loss of white matter integrity (Compston and Coles, 2008; Andriessen et al., 2010; Johnson et al., 2013; Tremblay, 2014; Armstrong et al., 2016). Since white matter integrity is essential for maintaining optimal activation of motoneurons during voluntary muscle contractions, we expect that mTBI will result in reduced voluntary activation of muscle fibers and affect measures of performance fatigability.

Therefore, it was the aim of the present study to assess the neuromuscular factors contributing to performance fatigability after mTBI, and to appraise these findings in the context of the increased levels of self-reported fatigue that occur post-injury. We hypothesize that mTBI may impair voluntary drive, thereby reducing muscle activation and leading to increased performance fatigability. Also, that this change might partially explain the increased levels of self-reported fatigue following mTBI. Furthermore, by investigating fatigue and fatigability using the same paradigm that was previously used in MS, we hope to increase the understanding of fatigue in neurological populations.

## Materials and Methods

### Study Population

Twenty-two patients with mTBI and 22 age- and sex-matched control participants were included. mTBI was defined by a Glasgow coma scale score between 13–15 on admission, with posttraumatic amnesia less than 24 h and/or loss of consciousness of less than 30 min. Inclusion criteria included persistent complaints of (self-reported) fatigue for more than 3 months post injury and adequate hand dexterity (to perform the motor tasks). Exclusion criteria included psychiatric disorder, neurologic disease (including previous TBI), and drug or alcohol abuse. Data from the control subjects were also used in an accompanying study (Sars et al., 2018). Experiments were designed in accordance with the declaration of Helsinki (World Medical Association, 2013) and approval of the experimental procedures was provided by the medical ethical board of the University Medical Center Groningen. Written informed consent was obtained from all participants before the experiment.

### Questionnaires and Cognitive Testing

For the mTBI patients, self-reported fatigue was quantified using two questionnaires: the Fatigue Severity Scale (FSS; Krupp et al., 1989) and the Modified Fatigue Impact Scale (MFIS; Multiple Sclerosis Council for Clinical Practice Guidelines, 1998; Schiehser et al., 2015). Mood was evaluated using the Hospital Anxiety and Depression Scale (HADS; Zigmond and Snaith, 1983). Cognitive impairment and attentional processing were assessed in mTBI participants using the written form of the Symbol Digit Modalities Test (SDMT; Smith, 1982) and the 3 s Paced Auditory Serial Addition Test (PASAT’3; Gronwall, 1977; McCauley et al., 2014), respectively. Handedness was evaluated using the Edinburgh inventory (Oldfield, 1971).

### Force Recording

**FIGURE 1:**
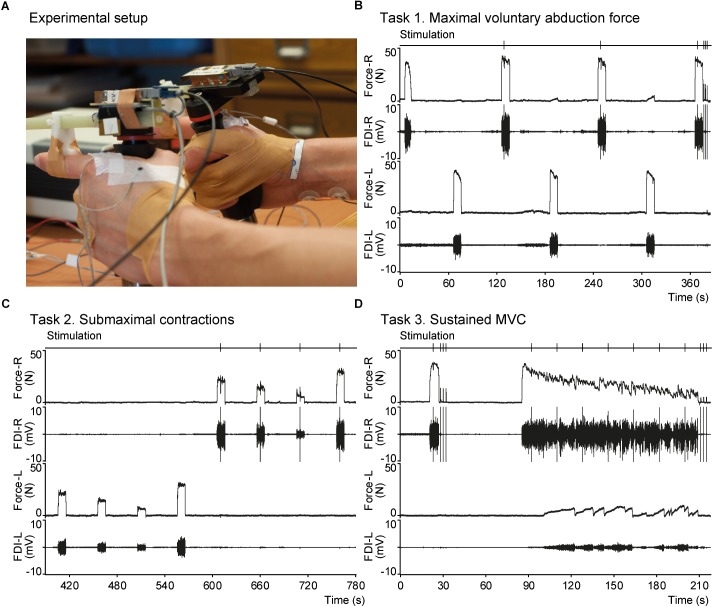
Experimental setup and an example of raw data. **(A)** Photograph showing the hands a participant equipped with force transducers. The finger bracket is positioned over the proximal interphalangeal joint of the index fingers as can be seen for the left hand. Placement of EMG electrodes over the FDI can be seen for the left hand and electrodes used for electrical stimulation can be seen on the right wrist. **(B–D)** Panels showing raw data recorded in a male mTBI participant during tasks 1–3, respectively. From top to bottom each panel shows the time-points of electrical nerve stimulation, index finger abduction force of the right hand, EMG of the right FDI, force of the left hand, and EMG of the left FDI. MVC, maximal voluntary contraction; FDI, first dorsal interosseous.

Index finger abduction force was measured using hand-held transducers (van Duinen et al., 2007; Figure 1A). Participants held the transducers with their index fingers extended. The horizontal bar of the transducer was aligned parallel to the index finger and the finger bracket was positioned over the proximal interphalangeal joint. The thumb was taped to digits III-IV to maintain this hand position throughout the experiment. Force signals was sampled at 500 Hz using a 1401 interface and recorded on a computer with Spike 2 software (version 7.12, Cambridge Electronic Design, Cambridge, United Kingdom).

### Electromyography

Surface EMG was recorded from the first dorsal interosseous (FDI) of both hands using sintered silver/silver chloride electrodes (In Vivo Metric, Healdsburg, United States). After cleaning the skin with alcohol, one electrode was applied over the muscle belly of each muscle and the second electrode was applied over the adjacent metacarpophalangeal joint. A reference electrode was applied over the right wrist. EMG signals were amplified (200 V/V) using custom-made amplifiers, band-pass filtered (10–1000 Hz), and sampled (at 2000 Hz) using the same interface as the force signals.

### Electrical Nerve Stimulation

Voluntary muscle activation of the right FDI was determined using the interpolated twitch technique (Merton, 1954; Behm et al., 1996; Gandevia, 2001). The ulnar nerve was electrically stimulated using a constant-current stimulator (pulse width 200 μs; DS7A, Digitimer, Welwyn Garden City, United Kingdom) via a pair of self-adhesive electrodes applied over the ulnar nerve close to right wrist. Stimulation intensity was set to ≥130% of the intensity required to evoke a maximal M-wave in the FDI (range: 39–100 mA). During the experimental tasks, forces were evoked using double pulses (10 ms interval) to improve the signal-to-noise ratio (Gandevia and McKenzie, 1988). The forces evoked at rest are referred to as doublet-force; to comply with previous literature, we use the term superimposed twitch (SIT) for the forces evoked during voluntary contractions.

### Motor Tasks

Participants performed three isometric motor tasks in which they were asked to abduct their index finger. Participants were seated behind a desk and could see their force production on a monitor in front of them during the tasks (see Figure 1).

#### Task 1 Maximal Voluntary Contraction (MVC)

Participants generated three maximal isometric index finger abductions (10 s) with the left hand and four with the right hand, alternating between hands. Contractions were followed by 50 s rest between subsequent contractions. A superimposed twitch was evoked during at least two maximal contractions with the right hand (∼3 s after the start of the contraction) and three doublet-forces were evoked at rest after the last contraction (2 s interval) to obtain potentiated doublet-forces.

#### Task 2 Submaximal Contractions

To determine differences in voluntary activation with a different method we also evoked superimposed twitches during submaximal contractions (De Serres and Enoka, 1998). Participants had to match their force level to a horizontal line indicating target force at 10, 30, 50, and 70% MVC. Three blocks were performed with each hand, alternating between the left and right hand (12 contractions per hand). Each contraction was sustained for 10 s with 40 s rest between contractions. A superimposed twitch was evoked during at least two sets of four contractions with the right hand.

#### Task 3 Sustained MVC

Participants generated a brief maximal index finger abduction for 6 s followed by 60 s rest. Next, participants generated a sustained MVC lasting 124 s. Superimposed twitches were evoked once during the brief MVC and at seven time points during the sustained MVC (18 s interval). Three doublet-forces (2 s interval) were evoked at rest after the brief MVC (doublet_pre_) and after the sustained MVC (doublet_post_).

### Analysis

Questionnaire and cognitive test scores were calculated. PASAT scores below the 5th percentile of the normative scores (i.e., <32/60 for ≤12 years of education and <35/60 for >12 years of education) were indicative of impairment (Rao et al., 1991). SDMT scores were expressed as a *T*-score of the normative data matched for age and education (Smith, 1982). FSS scores were indicative of fatigue if a participants scored greater than the cut-off of 4.5 (Flachenecker et al., 2002), and greater than 29 in the case of MFIS total scores (Schiehser et al., 2015).

Force and EMG data were processed in Spike 2; EMG signals were transformed by calculating the root mean square (rms) over a moving window of 500 ms. Maximal force and rms-EMG were determined for the MVCs. The largest of the three potentiated doublet-forces evoked at rest was taken as the reference value and the superimposed twitches evoked during the maximal and submaximal contractions in task 1 and 2 were expressed as a percentage of this force.

During the sustained MVC, force and rms-EMG at the start and the end of the sustained contraction were average over 6 s and expressed as a percentage of maximal force and rms-EMG, respectively. Furthermore, force and rms-EMG during the sustained MVC were averaged over 2 s epochs (62 epochs in total, the 7 epochs which overlapped the electrical stimulation were excluded from statistical analysis), and normalized to maximal force and rms-EMG obtained during the MVC (task 1). The coefficient of variation (CV_force_) for the right index finger abduction force was calculated by dividing the standard deviation during the 2 s epoch by the mean force. The superimposed twitches during the sustained MVC were linearly corrected for the decline in muscle force (due to factors within the muscle) using the following formula (Schillings et al., 2003):

$$\begin{array}{l} Corrected\;SIT\;at\;time\;t\\ =\frac{SIT_{\text{t}}}{doublet{\;}_{\text{pre}}-(time_{t}/124s)\times (doublet{\;}_{\text{pre}}-doublet_{\text{post}})}\\ \times 100\% \end{array}$$

with *t* as the time of stimulation, and doublet_pre_ and doublet_post_ as the doublet-force prior to and after the sustained contraction, respectively.

### Statistical Analysis

Statistical analysis was performed in R studio (R version 3.4.3). Normality was assessed graphically using quantile–quantile plots. Group comparisons of questionnaire scores and motor task parameters were performed by ANOVA with sex included as covariate in the analysis of motor parameters in order to better explain the remaining variance. Non-normally distributed data were transformed to obtain a normal distribution of the ANOVA residuals (Prescott, 2018). If transformation could not achieve a normal distribution of the model residuals, Mann–Whitney *U* tests were used. Values in the text are shown as mean (SD) for normally distributed data or median (range) for non-normally distributed data.

Multilevel modeling was used to analyze the superimposed twitches during the submaximal contractions (task 2). Pre-planned statistical models were used to describe the relationship between the size of the SITs and the force level at the time of stimulation. Next, group was added as a fixed effect to assess differences between mTBI and control participants, followed by sex.

To describe the changes over time, multilevel modeling was performed for the force, rms-EMG, and CV_force_ during the sustained MVC. Pre-planned statistical models were used to describe these time-related changes. We started with a simple model in which the intercept was allowed to vary randomly per participant. More complex models were constructed including time and the 2nd and 3rd degree polynomials of time as fixed effects, as well as random slopes for each of these variables per participant. After each step, statistical analysis determined whether the more complex model survived; the more complex model explained the data significantly better than the previous model if the Akaike information criterion (AIC) decreased by at least 2. For all parameters (force, rms-EMG, and CV_force_) the models including (2nd and 3rd) polynomials of time and random intercepts and slopes explained significantly more variance. Therefore, in the results section we only describe whether the more complex models in which group, sex, and interactions were added explained (significantly) more variance than this *basic model*. Next, group (mTBI) was added to the basic model as a fixed effect. Since sex and age can affect the time-related changes in force and EMG (Sars et al., 2018), we subsequently included these variables and their interactions to the model in a stepwise fashion. Model residuals were examined graphically for normality and heteroscedasticity (using quantile-quantile plots and heteroscedasticity plots) for all multilevel models. If required, the dependent variable was transformed to meet these criteria. Finally, robustness of the final models was tested by re-estimating the model on a trimmed dataset. This was done by first identifying the data points with scaled residuals greater than 2 for the corresponding model. These data points (model outliers) were then removed from the dataset and the model was re-estimated.

Associations between self-reported fatigue and the measures of performance and perceived fatigability were examined in mTBI participants. For this analysis, the MVC of the mTBI participants was expressed as a *Z*-score (calculated using the mean and standard deviation of MVCs in the control group, per sex). Associations were analyzed between force decline, voluntary muscle activation, and the change in doublet-force after the sustained MVC. Associations between two nominal variables were analyzed using Spearman’s rank test (e.g., FSS and HADS scores). Linear regression was used for the associations between continuous variables and for multivariate analyses. Model residuals were examined for normal distribution using quantile-quantile plots.

## Results

One mTBI participant had problems performing stable MVCs and the SIT deviated more than three SD from the mean. Another mTBI participant did not perform the sustained contraction adequately and showed progressively increasing force during the first minute. These two participants were excluded from all analyses. To maintain balanced groups, the two matched control participants were also excluded. The mean age of the remaining mTBI participants was 40.1 years (range: 23–56, 8 females) and 41.1 years in controls (range: 21–59, 8 females). One mTBI participant stopped contracting during the sustained MVC after 84 s. The data up to this time point were included in the analysis, however, this participant was excluded from all analyses regarding associations of self-reported fatigue and performance fatigability (we also checked whether inclusion of this subject affected our conclusion). Demographics and injury characteristics of the mTBI participants are shown in Table 1.

**Table 1:** Demographics and injury characteristics of mTBI participants.

	mTBI (*n* = 20)
Sex	
Male	12
Female	8
Mean age (years)	40.1 (10.9)
GCS on admission	
13	5
14	13
15	2
Mechanism of injury	
Fall	4
Traffic accident	10
Sports injury	4
Violence	1
Occupational injury	1
Median time since injury (months)	5 (3–18)
Education level^∗^	
4	5
5	4
6	10
7	1

*Data shown as n, mean (SD), or median (range). ^∗^Education level is provided on a Dutch 7-point scale (levels 5–7 refer to completion of different levels of higher education; for details see: Schaart et al., 2008). GCS, Glasgow Coma Scale.*

### Questionnaires

Participants with mTBI reported higher levels of fatigue and depression. The FSS scores were increased in mTBI compared to controls (5.2 ± 0.8 *SD* vs. 2.7 ± 0.8 *SD*, *P* < 0.01), and 15 out of the 20 mTBI participants scored greater than the cut-off of 4.5 indicative of fatigue (Flachenecker et al., 2002). The median MFIS score reported by the mTBI participants was 51 (range: 25–71); all but one participant scoring above the cut-off score of 29 for fatigue (Schiehser et al., 2015). Scores on the MFIS subdomains are shown in Table 2. Furthermore, scores on the depression domain of the HADS were higher in mTBI (median: 7, range: 2–14) than in control participants (median: 0, range: 0–2, *P* < 0.01).

**Table 2:** Questionnaire scores.

	Control (*n* = 20)	mTBI (*n* = 20)	*P*-value
FSS	2.8 (0.8)	5.2 (0.8)	<0.01^∗^
MFIS	–	51 (25–71)	–
MFIS physical	–	19.5 (5–32)	–
MFIS cognitive	–	27 (16–35)	–
HADS depression	0 (0–2)	7 (2–14)	<0.01^∗^
HADS anxiety	4 (0–7)	6 (4–12)	<0.01^∗^
Edinburgh inventory	85 (50–100)	90 (-100–100)	0.93

*Data shown as mean (SD) or median (range). ^∗^ Indicates statistically significant difference (P < 0.05). FSS, Fatigue Severity Scale; MFIS, Modified Fatigue Impact Scale; HADS, Hospital Anxiety and Depression Scale.*

### Cognitive Tests

The median score on the PASAT’3 was 49.5/60 correct responses (range: 31–59). One mTBI participant scored below the 5th percentile cut-off (i.e., <32/60 correct responses for an individual with <12 years of education; Rao et al., 1991). The median score on the SDMT was 51.5 (range: 36–72). Data were expressed as a *T*-score, corrected for age and education. The mean *T*-score was 41.7 (range: 30–75; Smith, 1982). Four participants had *T*-scores below the normal range (≤40); one of these participants fell in the category ‘very low’ (*T*-score ≤ 30).

### Baseline Measurements

Index finger abduction MVCs of the right hand did not differ between mTBI participants (40.5 ± 8.6 N) and controls (38.0 ± 11.2 N, *F*_1,37_ = 0.97, *P* = 0.33; with sex included as a covariate, see Table 3). Similar outcomes were observed for the left hand (Table 3).

**Table 3:** Motor parameters.

		Control (*n* = 20)	mTBI (*n* = 20)	*P*-value	
				Group	Sex
**Baseline parameters**				
MVC right (N)	M	43.3 (10.7)	44.8 (7.6)	0.33	<0.01^∗^
	F	30.0 (6.2)	34.0 (5.4)		
MVC left (N)	M	53.6 (13.0)	55.4 (9.0)	0.76	<0.01^∗^
	F	40.6 (8.0)	40.3 (5.6)		
Reference doublet-force (N)	M	11.3 (4.3)	13.9 (2.3)	0.06	0.14
	F	10.8 (2.5)	11.5 (1.8)		
MVC superimposed twitch (%reference)		2.8 (0.9–15.4)	3.9 (0.0–17.3)	0.78	
**Sustained MVC**				
Force first 6 s (% MVC)		81.8 (7.7)	80.8 (8.9)	0.71	
Force last 6 s (% MVC)	M	30.7 (10.1)	25.2 (9.3)	0.37	<0.01^∗^
	F	35.7 (9.2)	36.6 (6.7)		
Mean SIT (% reference; *n* = 7)		10.3 (1.9–31.0)	9.3 (2.2–32.9)	0.34	
Doublet_post_ (% reference)	M	40.1 (16.3)	33.7 (11.8)	0.32	0.18
	F	43.4 (10.4)	42.1 (11.4)		

*Data shown as mean (SD) or median (range). P-values are shown for group only or for group and sex if sex was a significant covariate in the ANOVA. ^∗^ Indicates statistically significant difference (P < 0.05). M, male; F, female; MVC, maximal voluntary contraction; SIT, superimposed twitch.*

**FIGURE 2:**
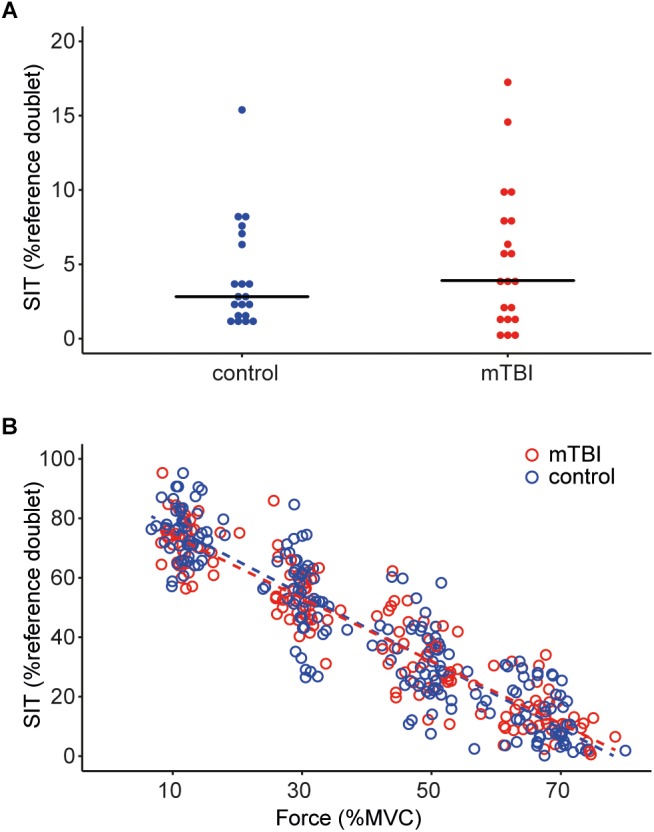
**(A)** Dot plot showing the size of the superimposed twitches during the maximal voluntary contractions in task 1, values are rounded to the nearest 0.5%. The horizontal line indicates the median for the control participants (blue dots) and mTBI participants (red dots). **(B)** Superimposed twitches evoked during the submaximal contractions (at 10, 30, 50, and 70% MVC) in task 2. Control data is shown in blue, mTBI in red. A linear regression line is shown for both groups, but no significant differences were observed between control and mTBI participants. SIT, superimposed twitch.

The potentiated doublet-forces at rest were also not different between mTBI (12.9 ± 2.4 N) and controls (11.1 ± 3.6 N, *F*_1,37_ = 3.91, *P* = 0.06, with sex included as a covariate, Table 3). The superimposed twitches during the brief MVC were also similar for the two groups (mTBI median: 3.9, range: 0.0–17.3 vs. control median: 2.8, range: 0.9–15.4% potentiated doublet-force, *F*_1,38_ = 0.08, *P* = 0.78; after logarithmic transformation. See Figure 2A).

### Voluntary Muscle Activation During the Submaximal Contractions

The superimposed twitches during the submaximal contractions were modeled using a multilevel model. The size of the superimposed twitches could be explained by a model including background force as fixed factor (β = -1.09, *t* = -42.18, *P* < 0.01) as well as random intercepts and slopes for force per participant. The model did not show significant improvement after including group or sex as fixed-factors. Thus, the twitch becomes on average 10.9% smaller when the voluntary background force increases with 10% MVC but we found no differences in the evoked twitches between controls and mTBI (see Figure 2B).

### Voluntary Force and rms-EMG During the Sustained MVC

During the sustained contraction the force during the first 6 s (control: 81.8 ± 7.7 vs. mTBI: 80.8 ± 8.9% MVC) and last 6 s (control: 32.7 ± 9.8 vs. mTBI: 30.0 ± 9.9% MVC) did not differ between groups (Table 3). A difference was observed, however, between males and females (*F*_1,36_ = 7.57, *P* < 0.01) indicating a larger force decline in male participants (Table 3).

**FIGURE 3:**
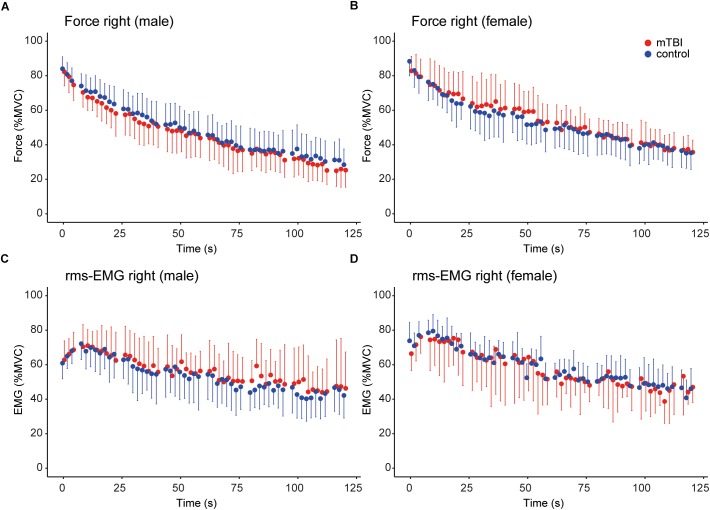
Time course of the force and rms-EMG of the right hand during the sustained MVC. For all panels, control data (*n* = 20) is shown in blue and mTBI (*n* = 20) in red. **(A,B)** Force (averaged over 2 s epochs) during the sustained MVC in male and female participants. **(C,D)** Mean rms-EMG (averaged over 2 s epochs) in male and female participants. Error bars in **(A–D)** show the standard deviation.

Instead of only using the data obtained at start and the end of the sustained contraction, we modeled the time course of the force. Adding group or sex to the *basic model* (see section “Materials and Methods”) did not result in a significant model improvement (group: Δ AIC = -1.9, sex: Δ AIC = -0.2). However, the model improved after including the interaction between sex and time (sex ^∗^ time: β = 4.968, *t* = -2.20, *P* = 0.03; Δ AIC = -2.7). No interaction was observed for time ^∗^ group. These results demonstrate that male participants showed a larger force decline over time, without a difference between mTBI and control participants (see Figures 3A,B).

The model explaining the variance of the rms-EMG of the right FDI did not improve after including group (Δ AIC = 2.0). However, the *basic model* improved significantly after including sex (Δ AIC = -2.6; sex: β = 2.10, *t* = 2.28, *P* = 0.03), indicating that rms-EMG was lower in male participants (see Figures 3C,D). Next, a significant interaction was found for sex ^∗^ group, but including this interaction did not significantly improve the model (*t* = -2.62, *P* = 0.01; Δ AIC = -1.9).

Finally, the coefficient of variation of the force of the right hand (CV_force_) was examined. To obtain a normal distribution of the residuals, CV_force_ was transformed using a power transformation (λ = 0.1). Including group, sex, and age in the *basic model* did not significantly improve the model. Thus, CV_force_ increased over time but no differences were found between groups or sex.

During the sustained contraction the contralateral, non-target FDI also becomes (unintentionally) active (Zijdewind and Kernell, 2001). Both contralateral force and rms-EMG were log transformed to improve distribution of the residuals. Both parameters increased over time and were best fit by the *basic model*, but no differences were observed between groups or sex.

Because one participants stopped the sustained MVC after 84 s, the multilevel analyses were repeated without this participant. This did not affect the conclusion for any of the parameters.

### Voluntary Muscle Activation During the Sustained Contraction

**FIGURE 4:**
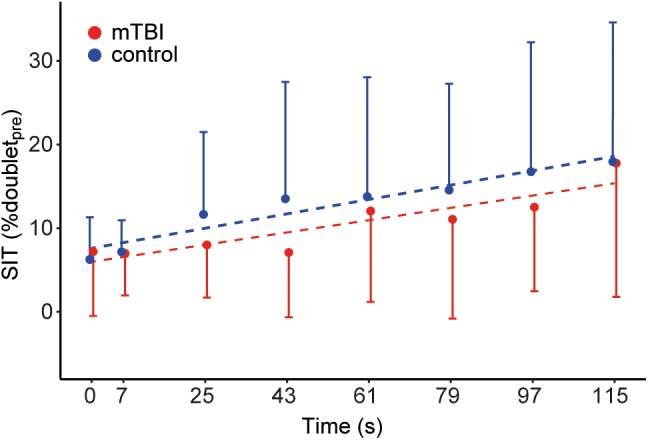
Time course of the superimposed twitches during the sustained MVC. Dots show the mean size of the superimposed twitches evoked before (*t* = 0 s) and during the sustained MVC (*t* = 7–115 s). Control data (*n* = 20) is shown in blue and mTBI (*n* = 20) in red. Error bars indicate the standard deviation. Superimposed twitches were linearly corrected for the decline in muscle force and expressed as a percentage of the doublet_pre_.

The time-course of the superimposed twitches before and during the sustained MVC) was modeled using a multilevel model. SITs were transformed by taking the square root. The model included time as fixed-factor (time: β = 0.99, *t* = 4.31, *P* < 0.01) and random intercept and slope for time per participant. The model indicates an increase in the size of the superimposed twitches over the course of the sustained contraction (i.e., a decrease in voluntary muscle activation), and no differences between mTBI and control participants (Figure 4). The mean of the seven superimposed twitches during the sustained MVC did not differ significantly between mTBI (median: 9.3 range: 2.2–32.9, *n* = 19) and control participants (median: 10.3, range: 1.9–31.0% doublet_pre_, *F*_1,37_ = 0.92, *p* = 0.34; after square root transformation of the mean SIT).

### Doublet-Forces After the Sustained Contraction

The doublet-force evoked after the sustained contraction declined in mTBI (to 37.2 ± 12.1% reference doublet-force, *n* = 19) and control participants (to 41.4 ± 14.0% reference doublet-force) but did not differ between groups (*F*_1,36_ = 1.03, *P* = 0.32; with sex included as a covariate).

### Associations Between Self-Reported Fatigue and Measures of Fatigability

Since mTBI participants reported significantly higher FSS scores than control participants, we analyzed associations between these questionnaires with different measures of fatigability for the mTBI participants only.

In mTBI participants (*n* = 20), no significant relationship was observed between the FSS scores and the HADS depression scores (*P* = 0.13). MFIS scores were, however, significantly correlated with HADS depression scores (rho = 0.73, *P* < 0.01); the association between HADS was also observed for the physical subdomain of the MFIS (rho = 0.70, *P* < 0.01), as well as the cognitive subdomain (rho = 0.52, *P* = 0.02). FSS or MFIS scores did not show significant associations with the raw PASAT or SDMT scores.

**FIGURE 5:**
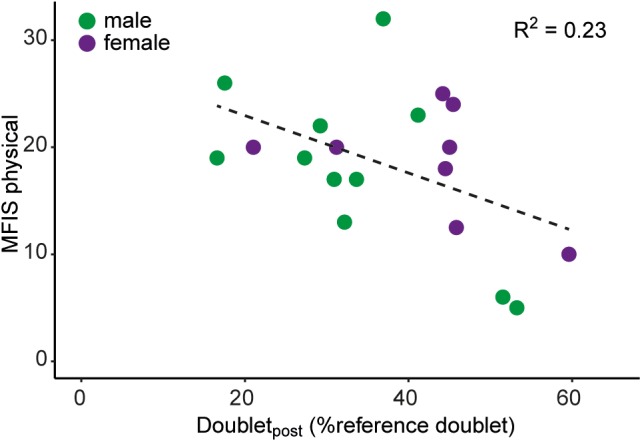
Association between scores on the physical subdomain of the MFIS and the decline in the evoked doublet-force after the sustained MVC (i.e., doublet_post_). Male mTBI participants (*n* = 11) are shown in green, female mTBI participants (*n* = 8) are shown in purple.

Linear regression of FSS or MFIS with force decline and MVC *Z*-scores (conform Steens et al., 2012) did not produce a significant association (FSS: *P* = 0.29; MFIS: *P* = 0.92, *n* = 19). However, a significant negative association was observed between the MFIS physical subscale and the decline in doublet-force (*R*^2^ = 0.23, *P* = 0.04, *n* = 19; Figure 5).

### Associations With Force Decline

**FIGURE 6:**
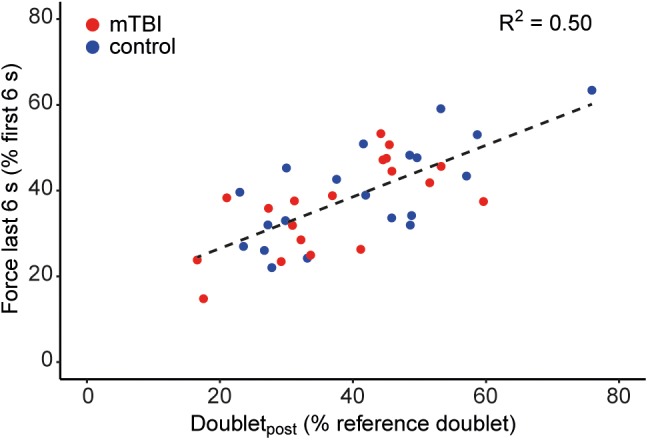
Association between force decline during the sustained MVC and the evoked doublet-force after the sustained contraction (i.e., doublet_post_) showing that participants with greater force decline also showed a greater decline in electrically evoked force. Control participants (*n* = 20) are shown in blue and mTBI (*n* = 19) in red.

Force decline (*n* = 39) during the sustained contraction (i.e., force during last 6 s/first 6 s) was negatively associated with the MVC (*R*^2^ = 0.17, *P* < 0.01) and positively associated with the decline in doublet-force (*R*^2^ = 0.50, *P* < 0.01; Figure 6). Force decline was not associated with voluntary muscle activation during the sustained MVC (i.e., mean SIT; *P* = 0.10). Analyzing force decline for the mTBI (*n* = 19) and control groups (*n* = 20) separately, the decline in doublet-force was able to explain more variance in control (*R*^2^ = 0.53, *P* < 0.01) than in mTBI participants (*R*^2^ = 0.45, *P* < 0.01). However, in mTBI participants significantly more variance (*P* = 0.03) could be explained by including voluntary muscle activation (mean SIT) in the model together with the decline in doublet-force (*R*^2^ = 0.59, *P* < 0.01; mean SIT: β = -4.03, *P* = 0.03, doublet_post_: β = 0.73, *P* < 0.01). Including the voluntary muscle activation was not able to improve the model in controls.

## Discussion

Consistent with literature (van der Naalt et al., 1999; Lundin et al., 2006; Stulemeijer et al., 2006; Lannsjö et al., 2009), participants with mTBI reported increased levels of fatigue and depression at more than 3 months post injury compared to control participants. No force or EMG differences were observed between groups during the sustained contraction reflecting similar levels of performance fatigability. Although individual differences in force decline could best be explained by changes in muscle properties (i.e., the decline in the doublet-force; see Figure 6), in mTBI participants the remaining variance could be explained by differences in voluntary muscle activation (i.e., mean SIT). Furthermore, (moderate) associations were found between scores on the physical subdomain of the MFIS questionnaire and the decline in doublet-force after the sustained contraction in mTBI participants.

### Fatigue and Performance Fatigability After mTBI

As expected, participants with mTBI reported increased levels of fatigue at more than 3 months post injury (van der Naalt et al., 1999; Lundin et al., 2006; Stulemeijer et al., 2006; Lannsjö et al., 2009); indicated by both FSS and MFIS scores. However, contrary to our expectations, performance fatigability in mTBI participants did not differ from age- and sex-matched controls.

The injury mechanism of mTBI has been well described. Impact-acceleration forces cause axonal injury and white matter tracts of the anterior corona radiata and corpus callosum are among the regions which are frequently damaged (Armstrong et al., 2016). Axon demyelination, as well as neuroinflammation with microglial activation develop post-injury (Andriessen et al., 2010; Armstrong et al., 2016). A post-mortem study using tissue from moderate-severely injured patients found that inflammation can persist for several years post-injury (Johnson et al., 2013). These findings are complemented by imaging studies in mTBI patients which have shown structural white-matter changes in addition to functional changes such as disruption of networks (Inglese et al., 2005; Eierud et al., 2014).

Force decline during a sustained maximal contraction provides a measure of performance fatigability (Enoka and Duchateau, 2016). This decline in voluntary force may occur due to changes in peripheral muscle properties; i.e., factors at and distal to the neuromuscular junction (Allen et al., 2008). In addition, muscle fibers can become less optimally activated by the central nervous system (Gandevia, 2001). The interpolated twitch technique can be used to provide an estimation of the portion of the muscle that is not maximally activated (Behm et al., 1996). Since maximal activation of muscle fibers requires optimal activation of the motoneuron pool we expected that voluntary muscle activation could be compromised after mTBI as the result of damage to white matter tracts and myelin modification that evolves post-injury (Inglese et al., 2005; Armstrong et al., 2016). This is especially important for hand muscles which receive a relatively large portion of synchronized input from the corticospinal tract (Day et al., 1989; Lemon, 2008; Keen et al., 2012). In a previous study, a small decline in voluntary muscle activation of the FDI was observed in the early stage following concussion (Powers et al., 2014). However, we did not find differences in force decline nor in voluntary muscle activation between mTBI and control participants in the present study.

Besides the force decline and voluntary muscle activation of the right FDI, we analyzed two other parameters as indirect measures of performance during the sustained MVC. Firstly, the coefficient of variation of the force of the right hand (CV_force_) was used as a measure of force steadiness. Secondly, the force and EMG of the contralateral (left) hand were recorded and analyzed. Both measures increase during fatiguing contractions (Lippold, 1981; Zijdewind and Kernell, 2001; Shinohara et al., 2003) and reflect increased effort. Neither parameter showed a difference between mTBI and control participants that could indicate increased effort in the mTBI group.

In both groups, individual differences in force decline during the sustained MVC were best explained by changes in peripheral muscle properties (i.e., the decline in doublet-force after the contraction; see Figure 6). However, only in the mTBI group could the variation in voluntary muscle activation (i.e., mean SIT during the sustained contraction) explain the remaining variance of the force decline. Although we were not able to objectify differences in performance between the groups, this may suggest that maintaining voluntary drive could be difficult after mTBI. This idea is further supported by the task performance of the two excluded participants and one mTBI participant who all had profound difficulties in performing the sustained contraction.

The rationale for investigating performance fatigability in the context of increased self-reported fatigue came from previous findings in individuals with relapsing remitting MS (Steens et al., 2012; Wolkorte et al., 2015a). Although the severity of fatigue reported by mTBI participants was similar to the levels of fatigue in MS (Steens et al., 2012: FSS 5.3 ± 0.9 *SD*), associations between self-reported fatigue and reduced voluntary muscle activation or (normalized) force decline were not found in the present study. However, a (moderate) association between the physical subdomain of the MFIS and the decline in doublet-force after the sustained MVC was observed in mTBI participants. The associations between the decline in doublet-force with self-reported (physical) fatigue as well as with force decline underline the importance of peripheral muscle properties to explain variation in fatigue and performance fatigability in persons with mTBI.

### Cognitive Impairment After mTBI

Cognitive impairment is a prominent complaint after mTBI (van der Naalt et al., 1999; Lundin et al., 2006; Lannsjö et al., 2009). In the present study we screened for cognitive impairment using the PASAT and SDMT. On both tests, performance of the mTBI participants was comparable to the reference population. It is possible that the 3 s PASAT task was not sensitive enough to capture subtle differences (Tombaugh, 2006), but also the SDMT showed low scores for only 20% of mTBI participants. Most of our mTBI participants (and controls) had completed a higher education program (see Table 1). Since higher education is known to attenuate changes in cognitive task performance due to TBI (Sumowski et al., 2013), it is possible that the relatively high level of education of our mTBI participants diminished the effects of the TBI on the cognitive (and perhaps also the motor) task.

Several studies have focused on the relationship between self-reported fatigue and performance during cognitive tasks; another example of performance fatigability (Ziino and Ponsford, 2006; Möller et al., 2014, 2017). For these tasks, also no associations between measures of self-reported fatigue and performance fatigability were observed.

### Fatigue and Depression

It is known that fatigue and depression are often found in the same patients and that fatigue and depression scores are mostly well correlated (Kluger et al., 2013). In the present mTBI population we found increased values for both depression and fatigue scores. We found an association between the HADS depression scores and the MFIS scores, but not the FSS scores. Though, previous studies have also reported the association between HADS depression scores and FSS scores after mTBI (Norrie et al., 2010; Möller et al., 2014).

### Force Measurements

Across all groups, the MVCs of the left hand were on average 20% larger than for the right hand. This difference has been reported before in our studies (e.g., Post et al., 2007) but not by other groups. Our force transducer is equipped with a full bridge strain gauge configuration and therefore records force independent of the position of the finger bracket (i.e., the connection between the index finger and the transducer; see Figure 1A; van Duinen et al., 2007). Furthermore, the force difference between the left and right hand remains present if the same force transducer is used for both hands. In other words, differences in the length of the index finger do not affect the force data and cannot explain the difference in force. In cadavers, we dissected three pairs of human hands to study the anatomical differences between the left and right FDI. No systematic differences were found between the left and right FDI for the mass, length, or cross sectional width; though the cross sectional width of the left FDI was larger in two pairs of specimens.

### Limitations of the Study

The target muscle of the present study was the FDI, a small hand muscle. We chose this muscle because we wanted to be able to compare the present data with the data obtained in persons with MS (Steens et al., 2012). Furthermore, intrinsic hand muscles are more strongly dependent on synchronized corticospinal input compared to most other muscles (Keen et al., 2012). Therefore, we expected differences in voluntary muscle activation in this muscle. It is, however, possible that measures of performance fatigability would have shown larger differences in other larger muscle (groups).

Contrary to most research related to fatigue following mTBI, we used motor tasks to investigate performance fatigability. A general limitation of using cognitive tasks to measure time-related changes (i.e., performance fatigability) is that performance on these tasks is often affected by learning processes. Since cognitive and motor tasks share higher cortical processes as illustrated by the direct interaction between cognitive and motor task performance in both controls (Lorist et al., 2002; Wolkorte et al., 2014) and clinical populations (Howell et al., 2013; Sarajuuri et al., 2013; Wolkorte et al., 2015b; Yasen et al., 2017), we expected to find differences between mTBI patients and controls in our motor tasks. An advantage of motor tasks is the ability to quantify output (force) continuously throughout the task. Furthermore, by using the interpolated twitch technique (Merton, 1954; Allen et al., 1994; Behm et al., 1996) during a sustained maximal muscle contraction it is possible to discriminate between factors residing in the muscle and factors in the central nervous system which are responsible for force decline. Nevertheless, the present study only found minor differences in motor parameters between controls and the mTBI population. It is possible that our outcomes measures were not sensitive enough and that a combination of cognitive and motor task would require more attentional resources and therefore induce larger differences.

## Conclusion

Fatigue is an important symptom after mTBI, but the underlying mechanisms remain poorly understood. Despite increased levels of self-reported fatigue, no differences were observed in force decline in persons with mTBI. Nevertheless, the present data indicate that changes in peripheral muscle properties could be important for understanding the mechanisms affecting perception of fatigue in persons with mTBI. These peripheral muscle properties together with changes in voluntary muscle activation were able to explain most of the variation in force decline during the sustained maximal contraction. Besides relevance for improving our understanding of fatigue, these findings also indicate the importance of studying a combination of motor and cognitive parameters after mTBI.

## Author Contributions

IZ and JvdN designed the experiments. RP and IZ performed the experiments and analyzed the data. RP, IZ, and JvdN wrote the manuscript.

## Conflict of Interest Statement

The authors declare that the research was conducted in the absence of any commercial or financial relationships that could be construed as a potential conflict of interest.
